# Prevalence of bovine trypanosomosis and assessment of trypanocidal drug resistance in tsetse infested and non-tsetse infested areas of Northwest Ethiopia

**DOI:** 10.1016/j.parepi.2017.02.002

**Published:** 2017-02-24

**Authors:** Shimelis Dagnachew, Biniam Tsegaye, Addissu Awukew, Meseret Tilahun, Hagos Ashenafi, Tim Rowan, Getachew Abebe, Dave J. Barry, Getachew Terefe, Bruno M. Goddeeris

**Affiliations:** a University of Gondar, FVM, P. O. Box 34, Gondar, Ethiopia; b Addis Ababa University, CVMA, P. O. Box 34, Debre Zeit, Ethiopia; c Global Alliance for Livestock and Veterinary Medicine, Scotland, UK; d Food and Agriculture Organization of the United Nations, FAO, Addis Ababa, Ethiopia; e University of Glasgow, CMVLS, 120 University Place, G12 8TA, Glasgow, UK; f KU Leuven, Faculty of Bioscience Engineering, 30 bus 2456, B-3001 Heverlee, Belgium

**Keywords:** Prevalence, Trypanosomosis, Tsetse, Trypanocidal drug resistance, Northwest Ethiopia

## Abstract

The Northwestern region of Ethiopia is affected by both tsetse and non-tsetse transmitted trypanosomosis with a significant impact on livestock productivity. The control of trypanosomosis in Ethiopia relies on either curative or prophylactic treatment of animals with diminazene aceturate (DA) or isometamidium chloride (ISM). In the present work; questionnaire survey, cross-sectional and experimental studies were carried out to; a) assess the utilization of trypanocidal drugs; b) determine the prevalence of bovine trypanosomosis and; c) assess the drug resistant problems respectively in Tsetse and non-tsetse infested areas on NW Ethiopia. A total of 100 respondents were included for the survey and the questionnaires focused on the drug utilization practices for the control of Trypanosomosis. Blood from cattle 640 (324 cattle tested in 2011, 316 cattle tested in 2012) and 795 (390 cattle tested in 2011, 405 cattle tested in 2012) were examined from tsetse infested and non-tsetse infested areas respectively using the buffy coat technique and thin blood smear for the detection of trypanosomes and measurement of packed cell volume (PCV). For the assessment of trypanocidal drug resistance three isolates, one from tsetse (TT) and two from non-tsetse (NT) areas were used on thirty six trypanosome naïve calves. The experimental animals were divided randomly into six groups of six animals (TT-ETBS2-DA, TT-ETBS2-ISM, NT-ETBD2-DA, NT-ETBD2-ISM, NT-ETBD3-DA and NT-ETBD3-ISM), which were infected with *T. vivax* isolated from a tsetse-infested or non-tsetse infested area with 2 × 10^6^ trypanosomes from donor animals, and in each case treated with higher dose of DA or ISM. The results of the questionnaire survey showed trypanosomosis was a significant animal health constraint for 84% and 100% of the farmers questioned in non-tsetse and tsetse infested areas of Northwest Ethiopia respectively. Responses on trypanocidal drug utilization practices indicated that risk factors for the development of drug resistance are common and treatment failures are frequently seen. Accordingly, the majority of farmers in tsetse infested area get trypanocides from drug stores and unauthorized sources whereas those from non-tsetse area get from veterinary clinics. Moreover, treatment administration is mainly by animal health personnel and treatment frequency is a maximum of three times/year/animal in non-tsetse area whereas it is administered mainly by the farmers more than seven times/year/animal in tsetse infested area. The prevalence of trypanosomosis varied from 17.59% in 2011 to 25.0% in 2012 in tsetse infested areas with a significant (*P* = 0.023) difference. Similarly, in non-tsetse infested area the prevalence was varied from 3.85% in 2011 to 5.93% in 2012 without significant rise. *Trypanosoma congolense* (75%) was the most prevalent followed by *T. vivax* (20.58%) and mixed infections (4.41%) in tsetse infested area while in non-tsetse infested area only *T. vivax* was detected. The overall mean PCV in parasitaemic animals (20 ± 2.3 SD) was significantly (*P* < 0.001) lower than that of aparasitaemic animals (27 ± 4.3 SD). The assessment of trypanocidal drug resistance tests revealed one isolate of non-tsetse infested area against DA in group NT-ETBD2-DA is resistant to the higher dose used with 3 relapsing animals (50% relapses) in the group. Another two relapses were detected one against ISM for the isolate from tsetse infested area (TT-ETBS2-ISM) and one against DA for another isolate (NT-ETBD3-DA) from the non-tsetse area. In conclusion, trypanosomosis is widely prevalent in both study areas causing significant reduction in the mean PCV values. Farmers' trypanocidal utilization practices appear to pose risks of drug resistance problems. The in vivo drug resistance tests indicated the presence of resistant parasites with the higher dose against DA for NT-ETBD2 isolate and suspected resistance problems were detected against ISM and DA for TT-ETBS2 and NT-ETBD3 isolates respectively. Therefore, trypanosomosis is a major constraint in Northwest Ethiopia and drug resistance is a threat in the control of trypanosomosis in both study areas.

## 1 Introduction

In Ethiopia, trypanosomosis is a serious constraint to livestock production and agricultural development. Due to the advancement of tsetse flies into formerly free areas, an estimated 220,000 km^2^ areas is presently affected by tsetse flies (Abebe, 2005). The most prevalent trypanosome species are *T*. *congolense* and *T. vivax*. Various authors reported that the prevalence of trypanosomosis in tsetse infested areas range from 11.85–37% (Rowlands et al., 1993, Abebe and Jobre, 1996, Dagnachew et al., 2005, Degu et al., 2012, Fikru et al., 2014). In non-tsetse infested areas of Northwest Ethiopia, the prevalence of trypanosomosis was in the range of 2–9% (Eneyew and Abebe, 1997, Cherenet et al., 2006, Sinshaw et al., 2006).

Trypanosomosis is controlled either by vector control or parasite control, or a combination of both. Parasite control currently relies on a small group of trypanocidal compounds, and new compounds are unlikely to become available in the near future (Barrett et al., 2004). Consequently, the use of these drugs must be carefully monitored and trypanosome populations need to be screened regularly for the appearance of drug-resistant parasites. There is increasing evidence that the efficacy of chemotherapy is becoming reduced by the widespread development of trypanosome drug resistance (Van den Bossche et al., 2000, Delespaux et al., 2008). Recent reports (Sow et al., 2012, Vitouley et al., 2012) confirmed ISM and DA resistance in *T. vivax* and *T. congolense* in West Africa. In Ethiopia the appearance of drug-resistant trypanosomes has been reported by several authors (Mulugeta et al., 1997, Afework et al., 2000, Tewelde et al., 2004, Shimelis et al., 2008a, Moti et al., 2012, Dagnachew et al., 2014). However, the reports on drug resistance are focused mainly on *T. congolense*. Therefore, this study was conducted with the aim of assessing risk factors for the development of trypanocidal drug resistance, determine the prevalence of bovine trypanosomosis and to test the efficacy of DA and ISM at higher doses against *T. vivax* from tsetse and non-tsetse infested areas of Northwest Ethiopia.

## 2 Materials and methods

**Fig. 1:**
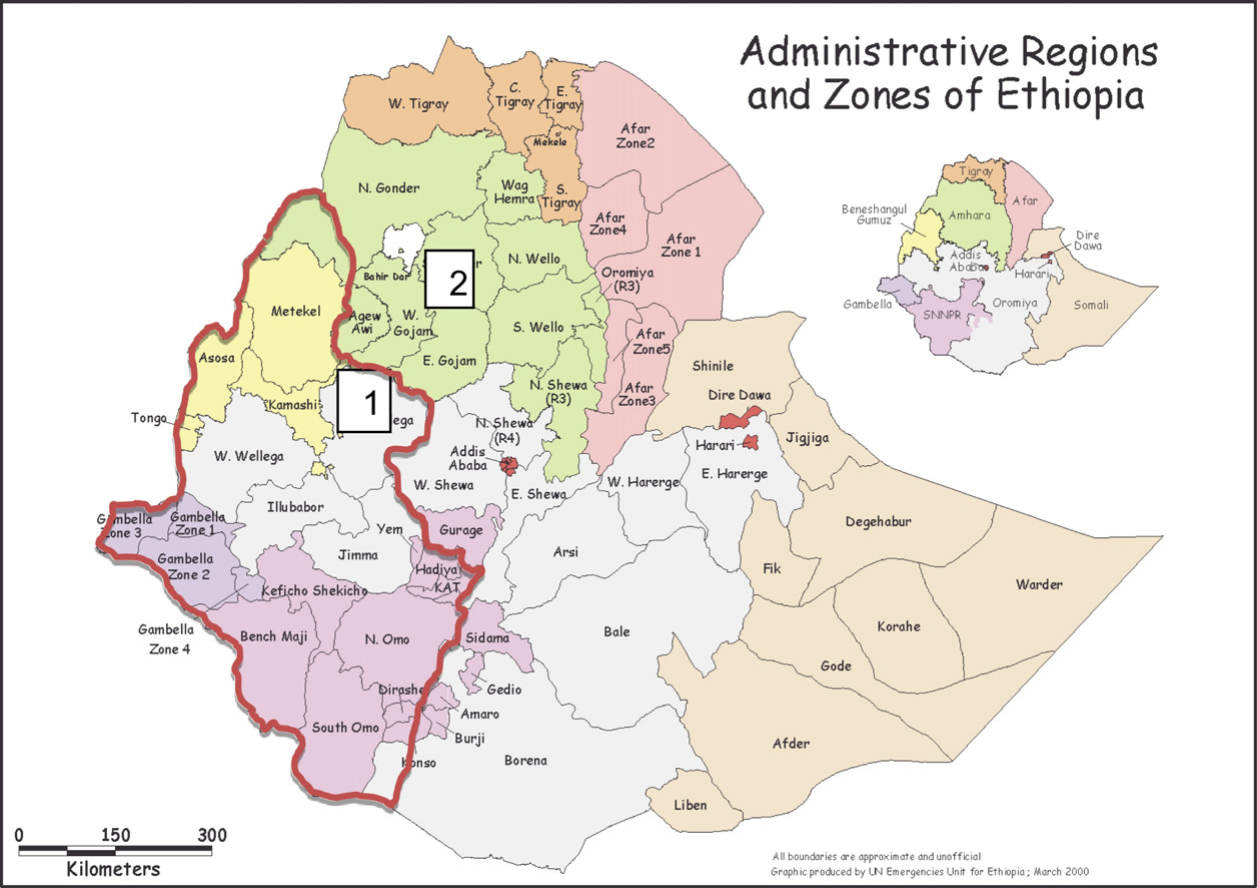
Map of administrative regions of Ethiopia and administrative zones of regions showing the study areas: tsetse infested area (Jabitehenan district of West Gojjam zone: 1) and non-tsetse infested area (Bahir Dar Zuria district of West Gojjam zone: 2). Tsetse infected areas of Ethiopia is encircled in red. (Source: Modified from World Food Program Vulnerability Analysis and Mapping Unit, Ethiopia, July 1998).

The questionnaire survey and prevalence were undertaken in two districts; from a tsetse infested district (Jabitehenan) and a non - tsetse infested district (Bahar Dar Zuria) in Northwest Ethiopia (Fig. 1). Jabitehenan is located between 10° 30′ 42″ N and 37° 7′ 11″ E with altitude range from 1100 to 1500 m above sea level (m.a.s.l.) whereas Bahir Dar Zuria is located 11° 36′ 0″ N and 37° 22′ 60″ E with altitude range from 1800 to 2000 m.a.s.l. The climate alternates with long summer rain fall (June–September) and a winter dry season (October–May) with mean annual rain fall of 1569.4 mm and the mean temperature varies between 16.7 °C - 31.6 °C. The livestock population includes cattle, sheep, goat and equines which are an integral part of the livelihood of the people. Cattle are particularly important in the agricultural activities where the farmers are dependent on oxen power for crop production (Central Statistical Agency (CSA), 2013). The assessment of trypanocidal drug resistance tests was conducted at Debre Zeit in the premises of College of Veterinary Medicine and Agriculture of Addis Ababa University.

### 2.1 Study methodology

#### 2.1.1 Questionnaire survey

A questionnaire survey was conducted to gather data on the problems of trypanosomes and practice of trypanocidal drugs usage in the study areas. The main component of the questionnaire focused during the interview includes; types of trypanocidal drug used, sources of drugs, treatment frequency and personnel's involved in the treatment practice of trypanosome cases etc. To address the questionnaire survey a total of 200 farmers 100 in each district were interviewed with a structured questionnaire format. The interviewed people were selected randomly from the study areas.

#### 2.1.2 Cross-sectional study

Cross-sectional studies were conducted in the months of October 2011 and 2012 to determine the prevalence of bovine trypanosomosis as well as measurement of packed cell volume (PCV) values between parasitaemic and aparasitaemic animals.

#### 2.1.3 Study animals

The study animals included indigenous Zebu cattle populations found in the two study districts. The husbandry system depends on natural grazing and crop residues and kept in a traditional communal management system. Animals obtain water in the rainy season from seasonal rivers while in the dry season from perennial rivers flowing long in their locality.

#### 2.1.4 Sampling method and sample size determination

A stratified multistage random sampling method was applied according to Thrusfield, (2007). Two districts were selected from Amhara National Regional State (first stage) to represent tsetse infested and non-tsetse infested areas of Northwest Ethiopia (Fig. 1). The lists of Peasant Association's 5PA's members within districts were compiled from data obtained in the district's agricultural office (second stage). Herds selected within the same grazing land were considered as strata and representative number of animals were sampled. Parameters like sex, age, sampling period and areas were recorded for each individual animal during sample collection. Age was categorized into two (< 2 years and > 2 years) groups. The sample size was determined based on the expected prevalence rate of 20% and 10% in tsetse infested and non-tsetse infested areas respectively, with an absolute desired precision of 5% at confidence level of 95%. Therefore, a total of 1435 cattle of which 640 (324 in 2011 and 316 in 2012) from tsetse infested area and 795 (390 in 2011 and 405 in 2012) from non-tsetse infested area were examined in the months of October.

#### 2.1.5 Parasitological examination and PCV determination

Paired blood samples were collected from auricular vein of each animal using two hematocrit capillary tubes that were filled 3/4 of the height and sealed with Cristaseal. The capillary tubes were centrifuged at 12000 rpm for 5 min first to measure the PCV. Thereafter, the capillary was cut 1 mm below the buffy coat to include the top layer of red blood cells, and the content of the capillary tube was expressed onto a clean slide, mixed and covered with a 22 × 22 mm coverslip (Paris et al., 1982). Then the slide was examined for trypanosomes based on their movement in the microscopic field. Confirmation of trypanosome species by morphological characteristics was done after staining with Giemsa by examination with oil immersion microscopy (Murray and Mcintyre, 1977).

#### 2.1.6 Isolation of Trypanosoma vivax parasites

Isolation of *T. vivax* parasites were carried out during 2012 study period for the assessment of experimental trypanocidal drug resistance tests. The isolates were collected from mono-infected animals with *T. vivax* using a parasitological technique (Paris et al., 1982) in the field. Stabilates were prepared from these animals at peak parasitaemia based on “rapid matching method” (Herbert, 1976). These stabilates were passaged into donor calves to see the typical motility and morphological structure of *T. vivax* using the parasitological technique and tested with PCR (Masiga et al., 1992) to confirm pure *T. vivax*.

#### 2.1.7 Assessment of trypanocidal drug resistance

##### 2.1.7.1 Experimental study site

The experimental study was carried out from March–June 2014 in a fly-proof animal facility in the premises of the College of Veterinary Medicine and Agriculture of Addis Ababa University at Debre Zeit, Ethiopia located at 9°6′N and 37°15′E with an altitude of 1920 m.a.s.l. about 47 km east of Addis Ababa.

##### 2.1.7.2 Experimental animals

A total of 36 indigenous Zebu (*Bos indicus*) cattle aged 9 to 12 months were purchased from a trypanosome free area. The animals were transferred into a fly-proof experimental animal house. Animals were ear-tagged, examined for the presence of trypanosomes and other blood parasites using blood smear technique and fecal egg count method (Paris et al., 1982, Soulsby, 1982) for helminthes. To avoid occurrence of pneumonia associated with transport stress and change of environment, each animal was treated on arrival with oxytetracycline 20% w/v (Chongqing Fangtong Animal Pharmaceutical Co., Ltd., China). All animals were treated with albendazole 2500 mg and Ivermectin at 0.2 mg/kg body weight (Chengdu Qiankum Veterinary Pharmaceuticals Co. Ltd., China) to control internal and external parasites. After treatment prior to the beginning of the experiment animals were acclimatized for one month for the new environment, handling and feeding conditions.

##### 2.1.7.3 Experimental design

The efficacy of higher doses of diminazene aceturate (DA) and isometamidium chloride (ISM) were tested against *T. vivax* isolates in experimentally infected cattle based on previous established protocols (Eisler et al., 2001). Animals were assigned randomly into six groups of six animals each: group TT-ETBS2-DA = infected with *T. vivax* isolate 2 from tsetse infested area and treated with DA, group TT-ETBS 2-ISM = infected with *T. vivax* isolate 2 from tsetse infested area and treated with ISM, group NT-ETBD2-DA = infected with *T. vivax* isolate 2 from non-tsetse infested area and treated with DA, group NT-ETBD2-ISM = infected with *T. vivax* isolate 2 from non-tsetse area and treated with ISM and group NT-ETBD3-DA = infected with *T. vivax* isolate 3 from non-tsetse infested area and treated with DA and group NT-ETBD3-ISM = infected with *T. vivax* isolate 3 from non-tsetse infested area and treated with ISM.

##### 2.1.7.4 Trypanosome challenge

Each experimental animal received 2 mL of infected blood from the donor animals at 10^6^ trypanosomes/mL via the intravenous route.

##### 2.1.7.5 Trypanocidal drugs

The trypanocidal drugs tested were diminazene aceturate (BERENIL R.T.U, Lot No.A189A01, Exp.03-2015, 20 Spartan Rd., Spartan, Republic of South Africa) and isometamidium chloride (Veridium T.M, Lot No.198A1, Exp. 06-2015, CEVA SANTE ANIMALE, Libourne-France) were tested. Diminazene aceturate was injected as a 7% solution at dose of 7 mg/kg body weight and isometamidium chloride was injected as 1% solution at dose of 1 mg/kg of body weight. Sterile distilled water was used to dissolve appropriate quantities of the drugs before it was administered to the animals. The drugs were administered through intramuscular route to animals on the basis of accurate body weight measurement taken immediately before treatment using digital weighing machine (TAL-TEC Livestock Scale, South Africa). All the trypanocidal drugs used for the experiment were tested for their quality (active ingredient) and fulfill certificate of the standards.

##### 2.1.7.6 Feeding and animal management

Animals were fed grass hay and supplemented with concentrates of wheat bran and green Elephant grass. Water and mineral lick were freely available. The handling of animals during the experiment was based on international guiding principles for biomedical research involving animals, as proposed by the Council for International Organizations of Medical Sciences (CIOMS), (1985). The research was authorized by the Animal Research Ethics Review Committee of the College of Veterinary Medicine and Agriculture of the Addis Ababa University (Permit No: VM/ERC/003/04/013).

##### 2.1.7.7 Trypanocidal drug resistance tests

After all experimentally infected animals of a group became parasitaemic they were treated with the higher doses DA or ISM. Starting from the treatment date, cattle were monitored for parasitaemia by the buffy coat technique twice a week for 100 days. When relapse (detection of trypanosomes after drug treatment) was detected the animal was treated with a second different drug.

### 2.2 Data analysis

Data was recorded during sample collection, parasitological examination and PCV measurement as well as questionnaire responses into Excel Spread Sheets to create a database and imported to SPSS version 20 for analysis. Descriptive statistics, Student's *t*-test and Logistic regression were used to explain results and compare variables. Logistic regression on the prevalence of trypanosomes was performed on the variables; sampling years, sampling areas, sex and age groups. Student's *t*-test was employed to compare the mean PCV of the parasitaemic animals with that of the aparasitaemic animals. For the drug resistance tests the interpretation was as follows; if relapse not was detected within 100 days after the first and the second trypanocidal drug administration, the treatment was considered successful and the trypanosomes sensitive to drug treatment. Relapse infections detected within 100 days of administration of a trypanocidal drug were taken as indicative of resistance. If relapse occurred in > 20% of the cattle tested, the strain was considered resistant to the dose of drug used (Eisler et al., 2001).

### 2.3 Results

#### 2.3.1 Questionnaire survey

**Fig. 2:**
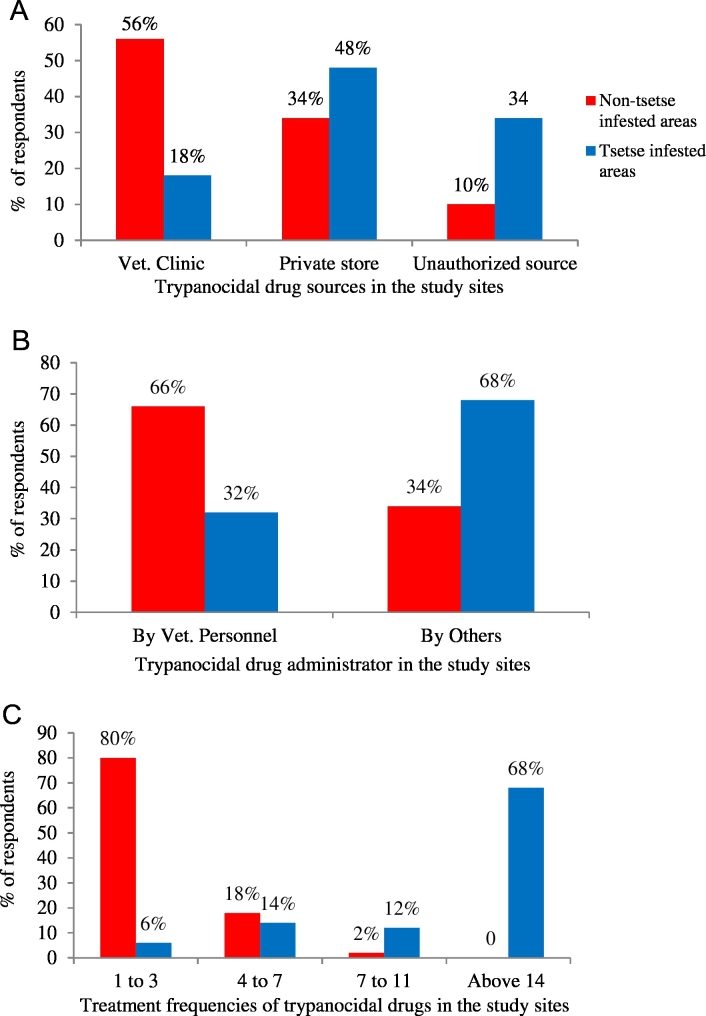
Questionnaire response on trypanocidal drug utilization practices in the study areas. (A) Sources of trypanocidal drugs for treatment, (B) administration of trypanocidal drugs in cattle, (C) trypanocidal drug treatment frequencies/animal/year.

All the respondents in tsetse infested and non-tsetse infested areas rear cattle primarily for draught purpose and income generation. Trypanosomosis was ranked as the first animal health constraint and known for the last two decades by 100% and 84% of the respondents from tsetse infested and non-tsetse infested areas respectively. Control of trypanosomosis in both areas relies mainly on treatment of animals with trypanocidal drugs; isometamidium chloride (ISM) and diminazene aceturate (DA). The vast majority of respondents (90%) from non-tsetse infested areas get trypanocidal drugs from veterinary clinics and drug shops whereas most respondents (82%) in tsetse infested areas ascertained that their drug sources were drug stores and unauthorized shops (Fig. 2A). On the other hand, most farmers in non-tsetse infested area send their cattle to veterinary clinics or animal health posts for trypanosomosis cases whereas majority of those in tsetse-infested area administer trypanocidal drugs by themselves or family members (Fig. 2B). According to the respondents, 40%, 20% and 40% of them use DA, ISM and both respectively in non-tsetse infested areas as compared to 2%, 30% and 68% in tsetse infested areas. However, the drug of their choice was DA in non-tsetse infested areas (52%) and ISM in the tsetse infested areas (94%). Eighty percent of respondents in tsetse infested areas treat their animals more than seven times/per year/animal whereas 80% of those in non-tsetse infested area give a maximum of three injections per year/animal (Fig. 2C). Trypanocidal treatment failures were reported by 66% and 94% of the respondents in non-tsetse and tsetse infested areas respectively.

#### 2.3.2 Parasitological findings

Out of the total 640 and 795 animals examined using parasitological techniques respectively from tsetse-infested and non-tsetse-infested areas, 21.25% and 4.91% (*P* < 0.001, 95%CI: 2.367–11.517) were found positive for the disease. In tsetse infested areas, the prevalence was significantly higher in 2012 compared to the finding in 2011 but no significant variation was observed in non-tsetse infested area (Table 1). Among the trypanosome species identified significantly higher prevalence was detected for *T. congolense* compared to *T. vivax* and mixed infections. Generally, the prevalence was significantly higher in male than female animals and in adults than young animals while such difference was not significant in the non-tsetse infested areas. When *T. vivax* was considered alone, the prevalence between tsetse infested (4.38%) and non-tsetse infested (4.91%) areas was not significantly different.

**Table 1:** Prevalence of bovine trypanosomosis in tsetse infested and non-tsetse infested areas of Northwest Ethiopia in October 2011 and 2012.

Parameters	Group	% Positive in 2011	% Positive in 2012	95%CI b/n years	
Parasite detection	Positive (tve) TT	17.59 (*N* = 324)^a^	25.0 (*N* = 316)^b^	0.437	0.939
	Positive (tve) NT	3.85 (*N* = 390)^c^	5.93 (*N* = 405)^c^	0.328	1.228
Parasite species	Tc TT	13.27 (*N* = 324)^a^	18.67 (*N* = 316)^b^	0.421	0.995
	Tv TT	3.70 (*N* = 324)^c^	5.06 (*N* = 316)^c^	0.309	1.436
	M TT	0.62 (*N* = 324)^c^	1.27 (*N* = 316)^c^	0.081	2.445
	Tv NT	3.85 (*N* = 390)^c^	5.93 (*N* = 405)^c^	0.328	1.228
Sex	Male tve TT	10.80 (*N* = 173)^a^	19.30 (*N* = 193)^b^	1.339	3.042
	Female tve TT	6.79 (*N* = 151)^a^	15.69 (*N* = 123)^b^		
	Male tve NT	2.11 (*N* = 194)^c^	2.96 (*N* = 196)^c^	0.577	2.091
	Female tve NT	1.79 (*N* = 196)^c^	2.96 (*N* = 209)^c^		
Age	Young tve TT	1.85 (*N* = 80)^a^	7.28 (*N* = 113)^b^	1.077	2.674
	Adult tve TT	15.74 (*N* = 244)^c^	17.72 (*N* = 203)^c^		
	Young tve NT	1.28 (*N* = 133)^a^	0.74 (*N* = 93)^a^	0.710	3.470
	Adult tve NT	2.56 (*N* = 257)^a^	5.19 (*N* = 312)^a^		

Tc-*Trypanosoma congolense*, Tv-*Trypanosoma vivax*, M-mixed (Tc and Tv), TT-tsetse infested area, NT-non-tsetse infested area, tve-positive. In each parameter of variables, superscripts with different letters indicate significant differences between values at *P* < 0.05.

#### 2.3.3 Isolation of *T. vivax* parasites

Isolation of *T. vivax* parasites were carried out in October 2012, consequently, 3 *T. vivax* isolates one from tsetse infested area and 2 from non-tsetse infested area were collected in stabilates forms. The stabilates were passaged into donor calves and develop infections and showed the typical motility and morphological structure of *T. vivax* using parasitological technique (Paris et al., 1982), and confirmed as pure *T. vivax* with PCR (Masiga et al., 1992). These stabilates were named as TT-ETBS2 for tsetse infested area and NT-ETBD2 and NT-ETBD3 for the non-tsetse infested area were used for the experimental drug resistance tests. The abbreviations indicated in the name of isolates stand as follows (TT - tsetse infested area, NT - non-tsetse infested area, ET - Ethiopia, BS - Birsheleko area of Jabitehenan district, BD-Bahir Dar Zuria district) i.e. *T. vivax* isolated from Ethiopia at Birsheleko areas of Jabitehenan district for tsetse infested area and at Bahir Dar Zuria district for non-tsetse infested area and the numbers are the sequential order of the isolates.

#### 2.3.4 Hematological findings

The hematological findings of the examined animals were investigated by comparing the PCV values with respect to trypanosome infection, species of trypanosomes, sampling sites and years. Consequently, overall mean PCV was significantly reduced in parasitaemic animals compared to aparasitaemic animals (*P* < 0.001) when samples were pooled from both study sites and study years. Similarly, irrespective of trypanosome status, mean PCV was significantly lower (*P* < 0.001) in tsetse infested area compared to non-tsetse infested area. However, the mean PCV values were not significantly different between sampling years (Table 2). The decrease in mean PCV was similar when data were sorted according to parasite species that the animal was harboring which was also true for those in non-tsetse areas compared to values for apparently aparasitaemic animals.

**Table 2:** Mean PCV of animals examined for trypanosomosis in both tsetse and non-tsetse infested areas of Northwest Ethiopia.

Parameters	Group	Number examined	Mean PCV	SD	*P*-value
Parasite detection	Positive	175	20	4.1	0.00
	Negative	1260	27	4.3	
Sampling years	2011	714	26	4.7	0.05
	2012	721	26	4.9	
Sampling areas	Tsetse area	640	25	5.4	0.00
	Non-tsetse area	795	27	4.2	

#### 2.3.5 Parasitemia and PCV values in experimental drug resistance tests

Clinical forms of trypanosomosis were observed until treatment was applied in all groups of experimentally infected animals. Establishment of infection was first detected within four days of challenge in animals infected with the non-tsetse isolate while on day seven for tsetse isolate infected groups. All infected animals became parasitaemic and treatment was given on day fourteen when they reached peak parasitaemia. Generally, isolates from non-tsetse infested area developed parasitaemia quickly as compared to the isolates from tsetse infested area.

**Fig. 3:**
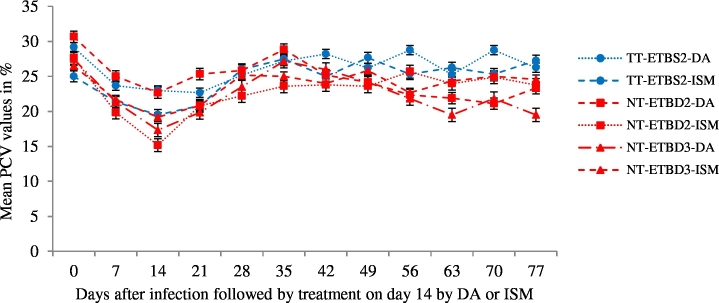
Mean ± SE PCV values before and after treatment either by DA or ISM in young Zebu cattle - six animals per group experimentally infected with *Trypanosoma vivax* isolates from tsetse (TT) and non-tsetse infested (NT) areas of Northwest Ethiopia, TT-ETBS2-DA and TT-ETBS2-ISM, NT-ETBD2-DA and NT-ETBD2-ISM, NT-ETBD3-DA and NT-ETBD3-ISM.

In all cases there was a significant reduction in the mean PCV values following infection with compared with before infection (*P* ˂ 0.05). Treatment of infected groups has resulted in significant improvements in the PCV compared to the values at the day of treatment (*P* < 0.05). However, the improvements were not significant particularly in relapse detected groups (Fig. 3). The comparison of mean PCV improvement between groups showed that animals in group NT-ETNBD2-DA and group NT-ETBD3-DA were found to show the least PCV improvement (*P* < 0.001).

#### 2.3.6 Drug resistance test

**Fig. 4:**
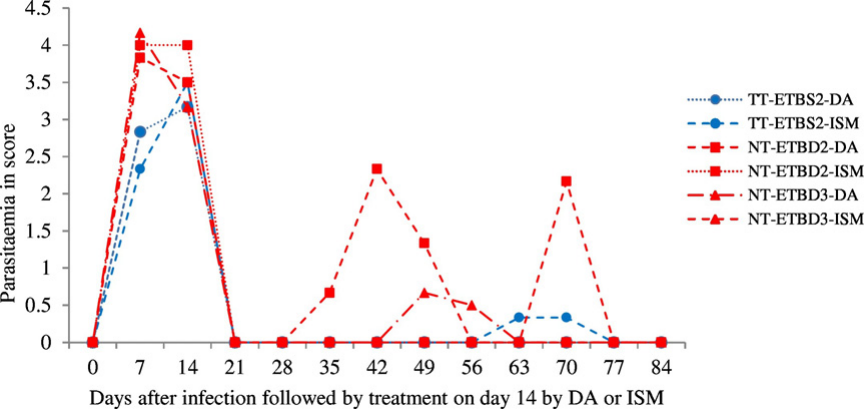
Mean parasitaemia in young Zebu cattle - six animals per group experimentally infected with *T. vivax* isolates from tsetse infested (TT-ETBS1 or TT-ETBS2) and non-tsetse infested (NT-ETBD1or ETBD2 or ETBD3) areas followed by treatment either by DA or ISM. It shows the mean of parasitaemia in all 6 animals prior to treatment, but the mean of only the relapsing 1–3 cattle days post treatment (dpt). From a total of six cattle in each group of NT-ETBD2-DA and NT-ETBD3-DA three and one animals showed relapse at 21 and 28 dpt respectively and from a total six cattle in TT-ETBS2-ISM group one animal showed relapse on 49 dpt.

Prior to treatment, peak parasitaemia was detected in all infected cattle. When the cattle were treated with DA or ISM, the parasitaemia was significantly reduced after 24 h. However, the assessment of drug resistance tests revealed the occurrence of resistant strains in cattle treated with the higher doses of both drugs. A total of five animals showed relapses; three from group NT-ETBD 2-DA) on 21 and 28 dpt, one animal from group NT-ETBD 3-DA) on 35 dpt and another one from group TT-ETBS 2-ISM) on 49 dpt. From the experiment, it was possible to conclude that *T. vivax* isolate (NT-ETBD2-DA) from non-tsetse infested area was found to be resistant at 7 mg/kg body weight of DA. The detection of two relapses one from another non-tsetse infested isolate for DA at 7 mg/kg body weight and one from tsetse infested isolate for ISM at 1 mg/kg body weight is indicative of the presence of resistant strains if more isolates were tested and improved diagnostic tests was used. The mean wave of parasitaemia in all 6 animals prior to treatment is shown, with the mean of the relapsing 1–3 cattle shown post treatment (Fig. 4).

### 2.4 Discussion

Bovine trypanosomosis and its impact is well known by the farmers in the study areas although the frequency of respondents was lower in non-tsetse area than in tsetse area. Similar observations have already been reported by Afework et al., (2000) from Metekel district of Northwest Ethiopia, Tewelde et al., (2004) from Western Ethiopia and Dagnachew et al., (2005) from Northwest Ethiopia where the cyclical vector tsetse fly are present. Similarly Zewdu et al., (2013) at Baro-Akobo and Gojeb river basins of Western Ethiopia reported that 94.1% of the respondents considered bovine trypanosomosis accounts for 64.6% of the total annual deaths of cattle in 2011/2012. To combat the problem, farmers reported to use diminazene aceturate (DA) and isometamidium chloride (ISM), the most commonly available trypanocidal drugs in the areas which are also similar for other places (Zewdu et al., 2013). The majority of the respondents from non-tsetse infested areas preferred to use DA whereas those in tsetse infested areas preferred ISM. A similar preference in non-tsetse infested areas has been reported by Van den Bossche et al., (2000) in the eastern province of Zambia. This could be linked to the fast treatment responses as in case of DA or longevity of protection from subsequent infections as in the case of ISM when given at a prophylactic dose.

Trypanocidal treatment frequencies are higher and injections were mainly given by farmers or untrained personnel in tsetse infested areas as compared to those in non-tsetse areas. This finding is in agreement with various reports done in Ethiopia (Afework et al., 2000, Tewelde et al., 2004, Shimelis et al., 2008b, Zewdu et al., 2013). Uilenberg (Uilenberg, 1998) reported that the number of treatments over a year reflects the magnitude of trypanosome challenge in an area. Moreover, most farmers in the tsetse area and significant number of them in non-tsetse area obtain trypanocidal drugs from drug stores and unauthorized sources without prescription. Altogether, farmers' practices suggest the occurrence of risk factors for emergence of trypanocidal drug resistance. Reasons for the misuse of trypanocidal drugs could be related to the inadequacies of veterinary services, the availability of trypanocidal drugs in informal markets and treatment failures that would force them to have the drugs at their back yard.

In the present study the prevalence of bovine trypanosomosis was 21.25% in tsetse infested areas and 4.91% was in non-tsetse infested areas which corroborate the report of Jemal and Hugh-Johns (Jemal and Hugh-Johns, n.d.) who reported trypanosomosis is a major constraint to the utilization of large land resources and also affect livestock, particularly cattle which have a major role in the agricultural economy of Ethiopia. Similar findings were also reported by different researchers (Afework et al., 2000, Tewelde et al., 2004, Dagnachew et al., 2005, Cherenet et al., 2006, Shimelis et al., 2008b). The higher prevalence of trypanosomosis in tsetse infested area compared to the non-tsetse area might be attributed to the dominance of *T. congolense* over *T. vivax*. Similar findings were reported in tsetse infested areas of Ethiopia, the dominant trypanosome species was *T. congolense* (Rowlands et al., 1995, Leak, 1999, Dagnachew et al., 2005). However, when animals infected with *T. vivax* alone were considered, prevalence was low and similar between tsetse infested and non-tsetse infested areas. This might be associated to *T. vivax* in these areas establish a more chronic infection and the conventional parasitological techniques might fail to detect the presence of the parasite in the blood. The lower prevalence reported for *T. vivax* is in agreement with various workers who reported a prevalence range of 2% to 9% (Eneyew and Abebe, 1997, Cherenet et al., 2006, Sinshaw et al., 2006).

Trypanosome infections were more prevalent in males and adult cattle than in females and young ones. This agrees with the following findings (Trail et al., 1994, Magona et al., 2008, Daya and Abebe, 2008, Dayo et al., 2010). The higher prevalence in male animals might be associated to the frequent exposure of male animals to the bites of vectors during traction period. The age difference could be linked to reproduction and work stresses to which most adult cattle are exposed for the bite of tsetse flies.

The fact that animal trypanosomosis causes significant anemia (Murray and Dexter, 1988, Dagnachew et al., 2005, Cherenet et al., 2006, Sinshaw et al., 2006, Degu et al., 2012) is supported by the present study. The fall in mean PCV was regardless of the trypanosome species detected in those parasitological positive animals. Moreover, very comparable lower PCV values were noticed in those animals infected with *T. vivax* from both study sites; altogether suggesting the pathologic significance of *T. vivax* in both tsetse and non-tsetse infested areas of Northwest Ethiopia.

The application of trypanocidal drugs is widely practiced to control trypanosomosis in domestic livestock since several decades either as curative or prophylactic drugs. This long time use of few trypanocides often predisposes the drugs to development of resistance. As indicated in the questionnaire survey, farmers' practices suggest widespread prevalence of risk factors for emergence of trypanocidal drug resistance. This has prompted us to undertake a controlled experimental trial to investigate the presence of trypanocidal resistance for *T. vivax* originated from both tsetse infested and non-tsetse infested areas of Northwest Ethiopia.

#### 2.4.1 Drug resistance tests

An earlier onset of parasitemia was detected in the NT infected cattle compared to the TT infected cattle. Furthermore, faster relapses of infection were also detected in the non-tsetse *T. vivax* origin. Anemia associated with trypanosome infections is multifactorial and the relative contribution of each mechanism will differ according to the host-parasite model, the phase of anemia development and the severity of infection. Rapid PCV recovery after treatment with trypanocidal drugs (Holmes and Jennings, 1976) was not observed in the present work. This might be due to the presence of drug resistant trypanosomes below the limits of our microscopical detection, though emergence of trypanosomes from drug inaccessible sites, nutritional imbalance and/or reduced response of the bone marrow due to exhaustion when the infection runs a chronic course cannot be ruled out (Murray and Dexter, 1988).

The presence of indiscriminate drug utilization practices and subsequent complaints over the efficacy of the available trypanocidal drugs in the present study areas was supplemented by the presence of resistant strains for both isolates of the drugs tested. This would indicate a drug resistance status for these two isolates of *T. vivax* against both DA and ISM, contrary to the view expressed by Fairclough, (1963), who suggested that it is difficult to induce resistance to ISM even by repeated low dosages (0.25–0.5 mg/kg body weight). Our previous experimental studies confirmed the occurrence of resistant strains of *T. vivax* against DA and ISM at 3.5 mg/kg and 0.5 mg/kg body weight respectively (Dagnachew et al., 2014). Based on this *T. vivax* isolates (TT-ETBS2, NT-ETBD2 and NT-ETBD3) from the two areas were tested using 7 mg/kg DA and 1 mg/kg ISM. Consequently, isolate NT-ETBD2 from non-tsetse infested area was found to be resistant to DA (three out of six animals relapsed). Codjia et al., (1993) reported resistance to the maximum recommended doses of DA (7 mg/kg body weight) for *T. congolense* isolates from cattle in the Ghibe valley Western Ethiopia. Moreover, in the present study the findings of two relapses, one from NT-ETBD3-DA group from non-tsetse infested area and one from TT-ETBS2-ISM group from tsetse infested area suggests the isolates are suspected for resistance to the two drugs and hence need further investigation. Similar findings were reported by Sow et al., (2012) indicating *T. vivax* isolated from Burkina Faso and tested at a dose of 1 mg/kg body weight for ISM was found to be resistant. As it has already been indicated in the epidemiological study, risk factors for prevalence of drug resistance are widespread in the areas from which the parasites were originated. Higher frequency of treatment, under dosage and mishandling especially when the drugs are administered by farmers and possible occurrence of poor quality drugs on the market could be reasons for the development of resistance (Vitouley et al., 2012). On the other hand, the difference in the drug resistance profile between different isolates of the parasite could be explained by the possible variation in exposure of the drugs to such risk factors (Holmes et al., 2004) and parasite variations in exposure to treatment.

In conclusion, the questionnaire survey underlined trypanosomosis is a major animal health constraint in both tsetse and non-tsetse infested areas of Northwest Ethiopia and trypanocidal drugs available in the area are exposed to risks of drug resistance. The cross-sectional studies conformed that bovine trypanosomosis is causing significant reduction in the mean PCV of parasitaemic animals in both areas. *T. congolense* and *T. vivax* were species of trypanosomes responsible for the occurrence of trypanosomosis in tsetse infested areas whereas only *T. vivax* species was identified in non-tsetse infested areas. Treatment of infected cattle with the recommend doses of trypanocides showed incomplete parasite clearance, consistent with the occurrence of resistant strains and adding to growing evidence that such resistance may be a problem. Therefore, appropriate control measures should be taken in both tsetse infested and non-tsetse infested areas of Northwest Ethiopia.

## Conflict of interest statement

The authors declare that they have no competing interests and have no any financial or personal relationships that could inappropriately influence or bias the content of the paper.
